# Arterial medial calcification (Mönckeberg’s sclerosis) with chronic renal disease in a zoo-kept Southern tamandua (*Tamandua tetradactyla*)

**DOI:** 10.1080/23144599.2021.1899408

**Published:** 2021-03-24

**Authors:** Tohru Kimura, Kengo Inaka

**Affiliations:** Laboratory Animal Science, Joint Faculty of Veterinary Medicine, Yamaguchi University, Yamaguchi, Japan

**Keywords:** Aortic calcification, arterial medial calcification, arterial mineralization, chronic renal failure, Mönckeberg’s sclerosis, Southern tamandua

## Abstract

Arterial medial calcification observed in animals is equivalent to Mönckeberg’s sclerosis in human beings. This lesion is rarely reported in domestic animals. In addition, little information is available concerning the occurrence of arterial medial calcification in zoo animals. The aim of the current work was to report arterial medial calcification incidentally encountered in a Southern tamandua (*Tamandua tetradactyla*). This paper described the clinical findings, haematological and serum biochemical profiles, and histopathological results. Haematological examinations showed decreases in white blood cell counts, erythrocytic parameters and platelets. In serum biochemical examinations, both of the creatinine and blood urea nitrogen levels markedly increased in this study. Glucose concentrations dramatically declined from the normal levels to the critical conditions. There was electrolytic imbalance which was not accompanied by increases in calcium and inorganic phosphorus concentrations.

Enzyme activities (ALP, AST, ALP, γ-GT, LDH and CK) remarkably increased just before the animal died. Histopathological examinations revealed that this marked and thickened calcification extended linearly around the circumference of the vessels. The calcified deposits were located entirely within the medial layer of the arterial wall. The lesions showed involvement of the internal elastic lamina with calcification. Severe calcification was observed in the glomerular capsules, indicating serious chronic kidney disease. We concluded that arterial medial calcification in the Southern tamandua arose with progressing chronic kidney failure.

## 1. Introduction

Atherosclerosis is the most common vascular disorder of human beings with important complications such as ischaemic heart disease and myocardial or cerebral infarction. The principal alteration is the accumulation of deposits (atheroma) of lipid, fibrous tissues and calcium in vessel walls, which eventually results in the blood vessels narrowing described in ruminants [1]. The lesion of the naturally occurring diseases has not found in domestic animals. It is known that Mönckeberg’s sclerosis is a manifestation of accelerated atherosclerosis in human patients with chronic kidney disease. Atherosclerosis medial calcification becomes prominent and has previously identified as Mönckeberg’s sclerosis. Arterial medial calcification (aortic calcification and arterial mineralization) observed in animals is equivalent to Mönckeberg’s sclerosis in human beings. This lesion is also rarely reported in domestic animals [2–9]. In contrast, little information is available concerning the occurrence of atherosclerosis and arterial medial calcification in zoo animals, excluding aged parrots [1].

Southern tamanduas have elongated snouts with tubular mouths that lack teeth and long tongues that are sticky with saliva when they feed. The animals are broadly distributed in the northern part of South America. Southern tamanduas in the wild subsist primarily on termites, ants and their larvae including high-quality protein, and occasionally eat other insects and ripe fruit. Their muscular stomach compensates for the lack of teeth and anteaters tend to show a low metabolic rate [10,11].

The aim of the current work was to report arterial medial calcification incidentally encountered in a Southern tamandua. This paper described the clinical findings, haematological and serum biochemical profiles, and histopathological results in this Southern tamandua.

## 2. Materials and methods

### 2.1. Animal

A 13-year-old male Southern tamandua weighing 4.2 kg was examined in Ube Municipal Tokiwa Zoo and Yamaguchi University. The animal presented with the onset of digestive signs such as weight loss, decreasing appetite, lethargy, vomiting, malabsorption, diarrhoea and alimentary tract hypermobility. The patient gradually lost weight and became emaciated, resulting in weight loss from 4.2 kg to under 3.1 kg in 6 months.

### 2.2. Blood sample collection

Blood was drawn under systemic anaesthesia using ketamine hydrochloride and medetomidine hydrochloride (i.m.). At 30 minutes after collection of blood samples, sera were separated by centrifugation at 1,500 × g for 10 minutes for biochemical analysis. For haematological samples, blood was collected into tubes containing K_2_EDTA.

### 2.3. Haematology and serum biochemistry

Complete blood counts and serum biochemical items including kidney and liver function tests were examined in this study. All of the parameters are shown in the footnote in Table 1.

**Table 1.** Haematological and serum biochemical findings in a Southern tamandua

Parameters	Units	The number of examinations			Reference values
		1	2	3	Mean ± SD or Range
Haematological findings					
WBC	×10^9^/L	16.2	9.3	3.3	8.07 ± 1.04
RBC	×10^12^/L	2.90	3.06	1.72	3.15 ± 0.23
Hb	g/L	98	91	49	107.3 ± 5.8
PCV	ratio	0.31	0.29	0.17	0.348 ± 0.015
MCV	fL	105.1	93.9	96.8	116.06 ± 7.46
MCH	pg	33.8	29.9	28.6	35.45 ± 2.05
MCHC	g/L	322	318	295	311.4 ± 16.5
RDW	%	13.6	17.6	18.1	―
PLT	×10^9^/L	192	357	310	―
MPV	fL	8.8	7.6	9.8	―
PCT	ratio	0.00169	0.0027	0.00305	―
PDW	%	16.7	13.6	21.7	―
Serum biochemical findings					
ALP	U/L	90	59	451	7–121
ChE	U/L	44	37	29	―
AST	U/L	46	69	953	13–65
ALT	U/L	34	39	91	48–98
γ-GT	U/L	57	176	198	18–225
LDH	U/L	179	87	900	0–329
LAP	U/L	53	68	64	―
AMS	U/L	131	78	35	―
Glu	mmol/L	5.72	3.50	0.50	2.66–6.77
CK	U/L	844	267	2000	0–757
BUN	mmol/L	34.8	50.00	50.00	7.14–12.14
Cre	μmol/L	228.8	449.9	716.8	―
UA	μmol/L	23.8	23.8	41.6	29.7–65.4
TP	g/L	71	67	52	8.49–9.33
ALB	g/L	28	29	23	24–34
A/G	ratio	0.65	0.76	0.79	―
T-Bil	μmol/L	5.13	5.13	25.66	0–3.42
T-CHO	mmol/L	4.25	4.40	1.48	2.51–9.14
TG	mmol/L	0.47	0.33	2.43	0.01–0.17
Na	mmol/L	128	138	132	136–144
K	mmol/L	5.5	4.6	14	4.3–5.9
Cl	mmol/L	86	96	104	98–108
Ca	mmol/L	2.50	3.55	3.30	9.7–13.5
IP	mmol/L	3.97	4.85	4.85	3.0–6.4
Mg	mmol/L	0.86	1.03	1.81	―
cCRP	nmol/L	57.1	N.D.	28.6	―
vSAA	μg/mL	16.3	N.D.	20.2	―

N.D.: not done

Reference values [22,23]

Haematological examinations

WBC: white blood cell count, RBC: red blood cell count, Hb: haemoglobin concentration, PCV: packed cell volume, MCV: mean corpuscular volume, MCH: mean corpuscular haemoglobin, MCHC: mean corpuscular haemoglobin concentration, RDW: red blood cell distribution width, PLT: platelet count, MPV: mean platelet volume, PCT: plateletcrit ratio and PDW: platelet distribution width.

Serum biochemical examinations

ALP: alkaline phosphatase, ChE: cholinesterase, AST: asparate aminotransferase, ALT: alanine aminotransferase, γ-GT: γ-glutamyl transpeptidase, LDH: lactate dehydrogenase, LAP: leucine aminopeptidase, AMS: amylase, Glu: glucose, CK: creatine kinase, BUN: blood urea nitrogen, Cre: creatinine, UA: urate, TP: total protein, ALB: albumin, A/G: albumin: globulin ratio, T-Bil: total bilirubin, T-CHO: total cholesterol, TG: triglycerides, electrolytes (Na, K, Cl, Ca and Mg), IP: inorganic phosphorus, cCRP: C-reactive proteins and vSAA: serum amyloid A.

### 2.4. Autopsy

In spite of careful medical treatment, the animal did not respond to therapy. After his death, we made an autopsy of this Southern tamandua.

### 2.5. Histopathology

Tissue specimens were obtained from the kidneys, and the thoracic and abdominal aorta. The tissue specimens were fixed in 10% neutral buffered formalin and 4-μm sections were stained with haematoxylin and eosine stain and by von Kossa’s stain, Weigert’s method and van Gieson’s method.

All procedures involving animals were approved by the Institutional Animal Care and Use Committee of Yamaguchi University (approval No. 409).

## 3. Results

### 3.1. Haematological findings and serum biochemical findings

Haematological profiles are shown in Table 1. The number of WBCs remarkably decreased in the course of this disease. The levels of erythrocytic parameters (RBCs, Hb concentrations and PCV ratio) were very low at the start of the examinations and these parameters subsequently remained unchanged. Mean corpuscular values (MCV and MCH) were maintained at considerably elevated levels during this study. The patient did not exhibit extremely reduced PLTs which were associated with clinical progression in this vascular disorder.

Serum biochemical profiles are shown in Table 1. Although Alb concentrations remained unchanged in this clinical course, TP concentrations gradually decreased, resulting in an elevated A/G ratio. UA concentrations increased approximately twice just before dying. Both of the Cre and BUN levels were markedly high in the first examination and these parameters rose even more markedly in the subsequent examinations. Glu concentrations dramatically declined from the normal levels to the critical conditions. Immediately before its death, T-CHO levels were reduced to a third of the initial result. In contrast, TG levels were rapidly raised 5–7 times as much as the result obtained from the first examination.

There were characteristic changes in the levels of serum electrolytes. Na and Cl concentrations were considerably low over the clinical course and K levels steeply rose just before dying. This patient developed stable measurements in the Ca and IP concentrations throughout this disease. Mg levels showed an upward trend during this investigation.

vSAA concentrations in the first and third examination increased more than 10 times those in healthy condition, while increased cCRP concentrations were found in the onset of this disease.

Enzyme activities (ALP, AST, ALP, γ-GT, LDH and CK) remarkably increased in the third examination carried out just before the animal died. In contrast, ChE and AMS activities gradually decreased during the course of this disorder.

### 3.2. Autopsy

At autopsy, the lung was diffusely congested and its surface had a gritty feel. The kidneys were markedly atrophic and had a pale granular surface. Affected arteries had diffusely rigid walls with marked loss of elasticity and showed solid, dense and pipe-like structures with raised, white and solid intimal plaques. The intimal surface of the entire aorta was rough, opaque and chalk-coloured. Diffuse thickening and calcification (calcified plaques) were clearly observed on the whole arterial walls. Artery walls appeared rough-surfaced, firm-to hard, plaques and coalescing nodules that narrowed the vascular lumens.

### 3.3. Histopathological findings

**Figure 1.**
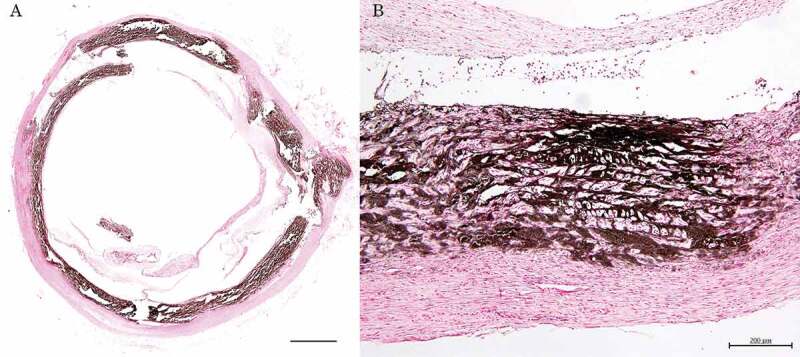
Mineralized lesions of the aorta

This marked and thickened calcification extended linearly around the circumference of the vessels. The calcified deposits were located entirely within the medial layer of the arterial wall. The aorta completely produced a concentric ring of medial mineralization. Mineralized lesions of the aorta showed complete encirclement of calcium deposition (Figure 1 (A)). The lesions showed involvement of the internal elastic lamina with calcification. Despite the widespread of this calcification, little inflammation was accompanied by these lesions.

A: Complete encirclement aorta with calcium deposition is seen. von Kossa’s stain. Bar = 1 mm.B: Marked disorganization and fragmentation of elastic fibres seen in the aortic media. Deposition of fibrin with erythrocytes and infiltration of neutrophils are observed in the tunica intima. von Kossa’s stain. Bar = 200 μm.

The aortic media showed marked disorganization and fragmentation of elastic fibres with aberrant collagen. There was also deposition of fibrin mixed with erythrocytes and infiltration of neutrophils in the intima (Figure 1 (B)).

**Figure 2.**
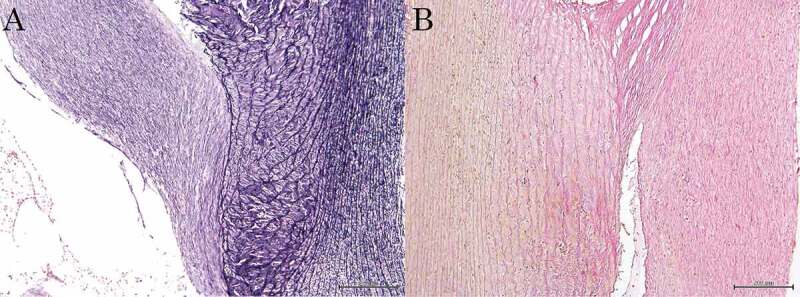
Microscopic photograph of the aorta

Fine to large granular calcification was found to deposit in vascular smooth muscle cells. Extracellular deposits were also observed in damaged and/or fractured internal elastic fibres (Figure 2 (A)). These bands of calcium-rich deposits thickened and became solid plates extending into the inner layer of the media. Large conglomerates of calcification formed solid plates, distorting the structure of the media. Subendothelial hyperplasia in the intima protruded into the vascular lumen. Smooth muscle cell necrosis, deposition of mucous substances and disruption of collagen fibres were found in the intima and the media. Severe calcification distorting the media spread over the entire circumference of the vessels (Figure 2 (B)). In contrast, neither intimal nor medial lipid deposition was observed in the arteries.

A: Fine to large granular calcification is found in vascular smooth muscle cells. Extracellular deposits are also observed in damaged and/or fractured internal elastic fibres. Weigert’s method. Bar = 200 μm. B: Subendothelial hyperplasia in the tunica intima is seen in the vascular lumen. Disruption of collagen fibres is found in the tunica intima and the tunica media. van Gieson’s method. Bar = 200 μm.

**Figure 3.**
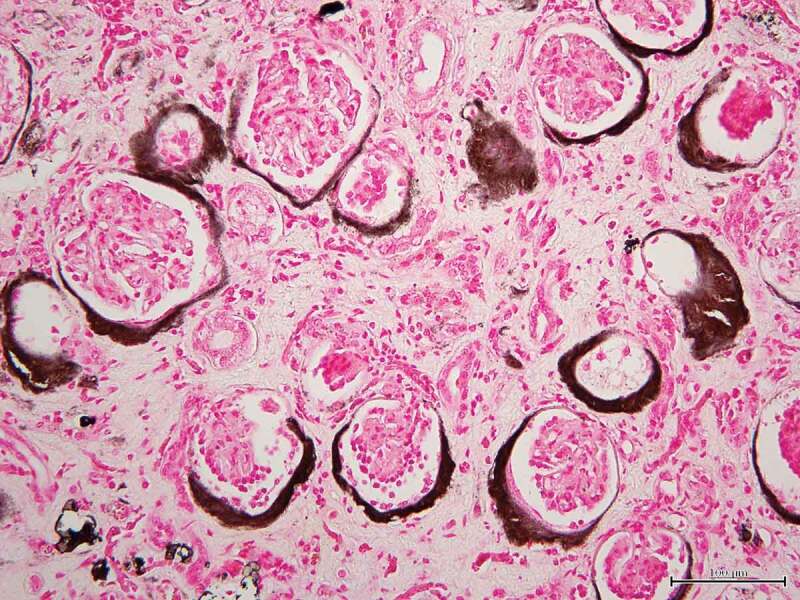
Microscopic photograph of the renal tissue

Severe calcification was observed in the glomerular capsules, indicating serious chronic kidney disease. Renal histopathological examinations readily detected glomerulosclerosis, glomerular atrophy, deposits of calcium and disrupted urinary tubules (Figure 3).

Severe calcification is observed in the glomerular capsules surrounding the glomeruli in crescent shape and stained black. von Kossa’s stain. Bar = 200 μm.

## 4. Discussion

Arterial medial calcification is an age-related disorder that occurs spontaneously in horses, rabbits and guinea pigs, and in rats affected with chronic renal diseases [1]. These conditions were certainly predisposed to arteriosclerosis in Southern tamanduas.

Progressive hypoleukocytosis was found on haematological examinations, indicating little possibility of infectious diseases. Decreases in erythrocytic parameters (RBCs, Hb concentrations and PCV ratio) represented an apparent progression of anaemia (microcytic and hypochromic anaemia). Increased RDW meant that erythrocytes were considerably of various sizes (anisocytosis). In contrast, there were not severe changes in the number and the distribution of platelets in the course of the disorder. Anaemic findings in this animal agreed with the results described in another report of a cat with chronic renal failure [6]. It was probable that this anaemia resulted from hepatic or renal dysfunction in erythropoiesis.

Although Alb concentrations remained clinically stable, TP concentrations gradually declined. Both of the BUN and Cre were kept at high levels, and then these parameters, including UA, remained elevated during this investigation. The findings revealed the critical features of chronic renal dysfunctions. There were no clinical signs such as jaundice in the terminal stage, even though Bil levels increased 5 times or more in the third examination. The present results from this Southern tamandua closely resembled those reported in previous studies in feline aortic sclerosis affected with chronic renal failure [6,7]. Glu concentrations fell precipitously in the third examination, suggesting unfavourable outcome.

T-CHO concentrations did not increase as compared with their reference values. The levels of lipid metabolic parameters (TG) were elevated in spite of the fact that this animal did not develop atherosclerosis. The changes in both parameters did not lead to the formation of arterial calcification.

Although there were unexpectedly no marked increases in Ca and IP concentrations, determination of electrolytes showed abnormal balances between Ca and IP concentrations. Other major changes in electrolytes were increased Mg concentrations in the third examination. These results were in agreement with the previous reports in feline cases [6,7]. These findings indicated an imbalance between absorption and excretion in the renal regulation.

Increases in vSAA concentrations suggested the presence of systemic inflammation for a long period of time. Increases in ALP, AST, ALP, γ-GT, LDH and CK activities were most likely caused by hepatocellular, skeletal muscular and renal damages. These findings reflected the advanced stage of arterial medial calcification with chronic renal failure. Increased enzymatic activities also accorded with our previous reports in the feline case [7].

Arteriosclerosis is defined as chronic arterial change consisting of hardening, loss of elasticity and luminal narrowing resulting usually from proliferative and degenerative changes of the media and intima. Inflammatory changes have little noticeable effects on the condition of the media and intima [12].

Aortic and cardiac mineralization was found in 21 of 3443 (0.61%) for dogs and in 1 of 786 (0.13%) for cats [8]. In human beings, the histopathological characteristic of atherosclerosis is fibrofatty plaque consisting of a raised focal plaque within the tunica intima, caused by the accumulation of lipid, lipid-laden foamy cells and covering fibrous cap [9]. It is believed that canine atherosclerosis has a mechanism of development indifferent from that of atherosclerosis in human beings. The theoretical reason is the formation of lesions originating from lipid deposition in the tunica intima and the infiltration of monocytes into the subendothelial space. Canine atherosclerosis is a rare consequence of altered lipid metabolism caused by hypothyroidism or diabetes mellitus [12]. A current medical concept contends that arterial medial calcification (Mönckeberg’s sclerosis) is a manifestation of accelerated atherosclerosis in patients with chronic kidney disease [13–21]. Canine aortic mineralization was probably explained by the developmental processes that resembled human lesions.

According to previous investigations, the etiologic relationship fundamentally exists between species and arterial medial calcification: human beings (accelerated atherosclerotic calcification with chronic kidney disease), dogs (accelerated atherosclerotic calcification related with hypothyroidism or diabetes mellitus), cats (close association with chronic renal failure), cattle (hypervitaminosis D_3_) and horses (siderocalcinosis: the result of deposition of both iron and calcium salts) [12].

Although Southern tamanduas originally live on insects, mainly termites (white ants), Southern tamanduas reared in the zoo are fed on ground meat mixed with cat foods. Longstanding alternative diet may cause chronic renal failure for this species and then the animal eventually developed severe arterial calcification. The pathogenesis of dietary arterial calcification in Southern tamanduas was similar to, if anything, that reported previously in a case of feline aortic sclerosis [7]. It is stated that human extensive mineralization of the aorta occurs as the result of hypercalcaemia and/or hyperphosphatemia. Our results revealed that arterial calcification in the present case was not associated with increased IP and Ca concentration in serum resulting from severe chronic renal failure. Anteaters such as Southern tamanduas have a metabolic rate that is lower than those of other mammals [10,11]. This low metabolic rate provides insight into slow disorder processes and the occurrence of remarkable arterial medial calcification. It was probable that the low metabolism in Southern tamanduas slowly caused calcium deposition in the vessels on the basis of a disproportion between Ca and IP concentrations.

## 5. Conclusion

To the best of the author’s knowledge, in the case of a Southern tamandua, we reported for the first time the occurrence of arterial medial calcification with severe chronic renal failure. Our study revealed that there was a difference in the pathogenesis of arterial medial calcification (Mönckeberg’s sclerosis) between human beings and insectivorous tropical American mammals (Southern tamanduas). We concluded that their arterial medial calcification arose with progressing chronic kidney disease, whether atherosclerosis and clinical pathological changes (hypercalcaemia and/or hyperphosphataemia) were present or absent.
